# Microbial community structure and metabolic characteristics in sediments from five different deep-sea trenches

**DOI:** 10.3389/fmicb.2025.1676738

**Published:** 2025-12-03

**Authors:** Yao Xiao, Hao Liu, Ziying Wu, Xuegong Li, Hongmei Jing

**Affiliations:** 1 State Key Laboratory of Deep-Sea Science and Intelligent Technology, Institute of Deep-sea Science and Engineering, Chinese Academy of Sciences, Sanya, China; 2 University of Chinese Academy of Sciences, Beijing, China; 3 HKUST-CAS Sanya Joint Laboratory of Marine Science Research, Chinese Academy of Sciences, Sanya, China

**Keywords:** community structure, production rate, respiration rate, growth efficiency, trenches

## Abstract

Microbial community growth efficiency, defined as the ratio of production to substrate assimilation, could provide insights into carbon flow among microbes and the regulation of marine biogeochemical cycles. However, the metabolic characteristics of microbes in deep-sea trenches remain largely undetermined. In this study, the structural and metabolic characteristics of microbial communities in five deep-sea trenches were investigated using Illumina high-throughput sequencing, quantitative PCR, the ^3^H-leucine incorporation method, and electron transport system analysis. We found that microbial community structure and functional gene abundance exhibited significant inter-trench variations, indicating that geographic isolation and environmental filters are key drivers of microbial biogeography. Under atmospheric pressure (AP), significantly higher respiration rates in the Mariana (MT) and Yap (YT) trenches than in the Kermadec (KT), Diamantina (DT), and Wallaby-Zenith (WT) trenches showed that higher organic carbon input in the western Pacific supported more active heterotrophic metabolism. Crucially, the consistently lower prokaryotic growth efficiency (PGE) under high hydrostatic pressure (HHP) across all trenches indicated that, *in situ*, pressure fundamentally shifted carbon allocation from biomass production to maintenance respiration, drastically constraining deep-sea carbon conversion efficiency. This demonstrated that genomic potential alone was insufficient to predict carbon cycling rates, and that direct physiological measurements under *in situ* conditions were essential for accurate assessment. Our study provided preliminary insights into the processes and efficiency of microbial-driven carbon cycling in the deep biosphere.

## 1 Introduction

The deep-sea environment is characterized by near-complete darkness, high hydrostatic pressure, low temperatures, and limited availability of organic matter (Jørgensen and Boetius, 2007). The deep-sea microbial food web is fundamentally dependent on the flux of particulate organic carbon from primary production in the euphotic zone (Nagata et al., 2010). Previous discoveries challenge the long-held view that the cycling of organic matter is slow in the deep sea and that microbial food webs in this environment are static in structure and function (Nagata et al., 2010). Data showing spatial variation in prokaryotic abundance and activity support the hypothesis that deep-sea microorganisms respond dynamically to variations in organic matter input to the bathypelagic realm (Nagata et al., 2010). Although the deep sea supports a diverse array of prokaryotes, the assimilation and transformation of natural carbon sources by these organisms remain poorly understood. Therefore, a systematic investigation of the composition, distribution, and metabolic status of microbial groups is fundamental to the ecological functioning of these unique deep-sea ecosystems.

A number of surveys using 16S rRNA gene amplicon sequencing have revealed the remarkable diversity of microbial communities present in deep-sea sediments (Jing et al., 2022; Zhang et al., 2024). These microbial communities exhibit unique metabolic properties and play essential roles in global biogeochemical cycles. However, knowledge of the carbon metabolic rates of microbial communities in deep-sea sediments remains limited. Prokaryotic growth efficiency (PGE), defined as the ratio of prokaryotic respiration (PR) to production (PP), is a proxy for prokaryotic carbon metabolism that evaluates the fate of organic inputs in aquatic systems (del Giorgio et al., 1997). The measurement of microbial metabolic traits enables the determination of the amount of carbon required for life, thereby contributing to quantitative biogeochemical studies. It is already known that an enhanced supply of dissolved organic carbon (DOC) and nutrients could increase microbial growth and stimulate bacterial activity (Yuan et al., 2010), and increased organic matter deposition in benthic sediments would enhance microbial activity and result in high microbial carbon conversion rates in the Mariana Trench (Glud et al., 2013; Jing et al., 2022). Pressure has a significant impact on the heterotrophic prokaryotic enzyme activities in deep-sea sediments (Xiao et al., 2021; Li et al., 2025). Nevertheless, research on the effects of pressure on microbial carbon activity in hadal trenches remains comparatively limited (Røy et al., 2012).

Hadal trenches, the deepest oceanic regions with extremely high hydrostatic pressure (e.g., > 60 MPa) and isolated hydrotopographical conditions (Jamieson et al., 2010), generally host a diversity of hadal life with a high degree of endemism and density (Jamieson et al., 2010). Geological and physicochemical conditions are highly varied across inter- and intra-hadal trenches. The carbon source in the hadal zones mainly results from the downward transportation of surface sediments via the funnel effect of trench geomorphology (Gamo and Shitashima, 2018; Bao et al., 2018). The preferential export of recalcitrant dissolved organic carbon through the microbial carbon pump leads to an enrichment of recalcitrant components in the sedimentary organic carbon on the trench seafloor (Jiao et al., 2010). Our previous EcoPlate cultivation experiments showed that trench microorganisms preferentially consume polymers, followed by carbohydrates, amino acids, and carboxylic acids (Wang et al., 2022). Furthermore, the presence of an abundant and diverse community of hydrocarbon-degrading microbes in the Mariana Trench indicates that the benthic microbial community is primed to break down complex organic compounds (Zhong et al., 2020).

A recent breakthrough by Xiao et al. (2025), using metagenomic sequencing, revealed a large number of novel species, whose community assembly was predominantly driven by homogeneous selection and dispersal limitation, consequently leading to distinct carbon metabolic strategies. Homogeneous selection favored streamlined taxa specialized in the efficient degradation of recalcitrant aromatic compounds, whereas dispersal limitation promoted metabolically versatile taxa capable of utilizing diverse carbon sources. Previous studies have demonstrated that different microbial communities prefer different organic matter sources, which in turn significantly influence their metabolic activities and community composition (Kenarova et al., 2013; Kumar et al., 2019; Wang et al., 2022; Liu and Jing, 2024). Although the rich microbial genetic potential for carbon degradation was identified in hadal metagenomic surveys, the actual *in situ* microbial metabolic rates and carbon conversion efficiencies, especially under high pressure, remain largely unknown.

In this study, we focused on five trenches, each characterized by unique geological and physicochemical properties. The Kermadec Trench (KT) reaches a maximum depth of 10,047 m and is located approximately 120 km off the northeastern coast of New Zealand (Angel, 1982). The Diamantina Trench (DT), located approximately 1,500 km west of Perth, Australia, in the Indian Ocean, has a maximum depth of approximately 8,047 m (Stewart and Jamieson, 2019). The Wallaby-Zenith Trench (WT), which extends from the continental margin of Western Australia, runs northwest for approximately 2,000 km and represents a structurally complex area of rugged topography composed of numerous plains, troughs, and ridges (Gibbons et al., 2012; Bond et al., 2023).

Although these three trenches are all located in the southern hemisphere near Australia, they span distinct biogeographic and environmental gradients. For instance, KT lies in the Southwest Pacific and is influenced by subduction-related geology and Antarctic Intermediate Water, whereas DT and WT, situated in the Indian Ocean, are shaped by different tectonic histories and oceanic circulation patterns (Stewart and Jamieson, 2019; Bond et al., 2023). On the other hand, the Yap and Mariana trenches (YT and MT), formed by plate collision, are both located in the western Pacific Ocean and are characterized by active plate convergence and hydrodynamic interconnection (Crawford et al., 1986). The southern MT intersects the north–south trending YT, facilitating water exchange that influences local biogeochemistry and microbial dispersal (Crawford et al., 1986). This configuration offers a unique opportunity to examine microbial divergence under shared regional hydrography but distinct trench morphologies. Among the five trenches, variations in microbial diversity and associated biogeochemical activities have been revealed in different trench sediments (Jing et al., 2022; Sun et al., 2024; Zhang et al., 2024), but their microbial metabolic characteristics are largely unknown (Wit et al., 1997; Røy et al., 2012). Given the highly varied geological structures and physicochemical conditions across different hadal trenches, it is necessary to elucidate the composition and metabolic characteristics of microbial communities across multiple trenches.

The present study investigated the diversity, community composition, and metabolic characteristics of microbes in sediment collected from five trenches to elucidate the geographical distribution and metabolic features of microbial communities across trenches, as well as the underlying mechanisms responsible for potential discrepancies.

## 2 Materials and methods

### 2.1 Sample collection and chemical analysis

**Figure 1:**
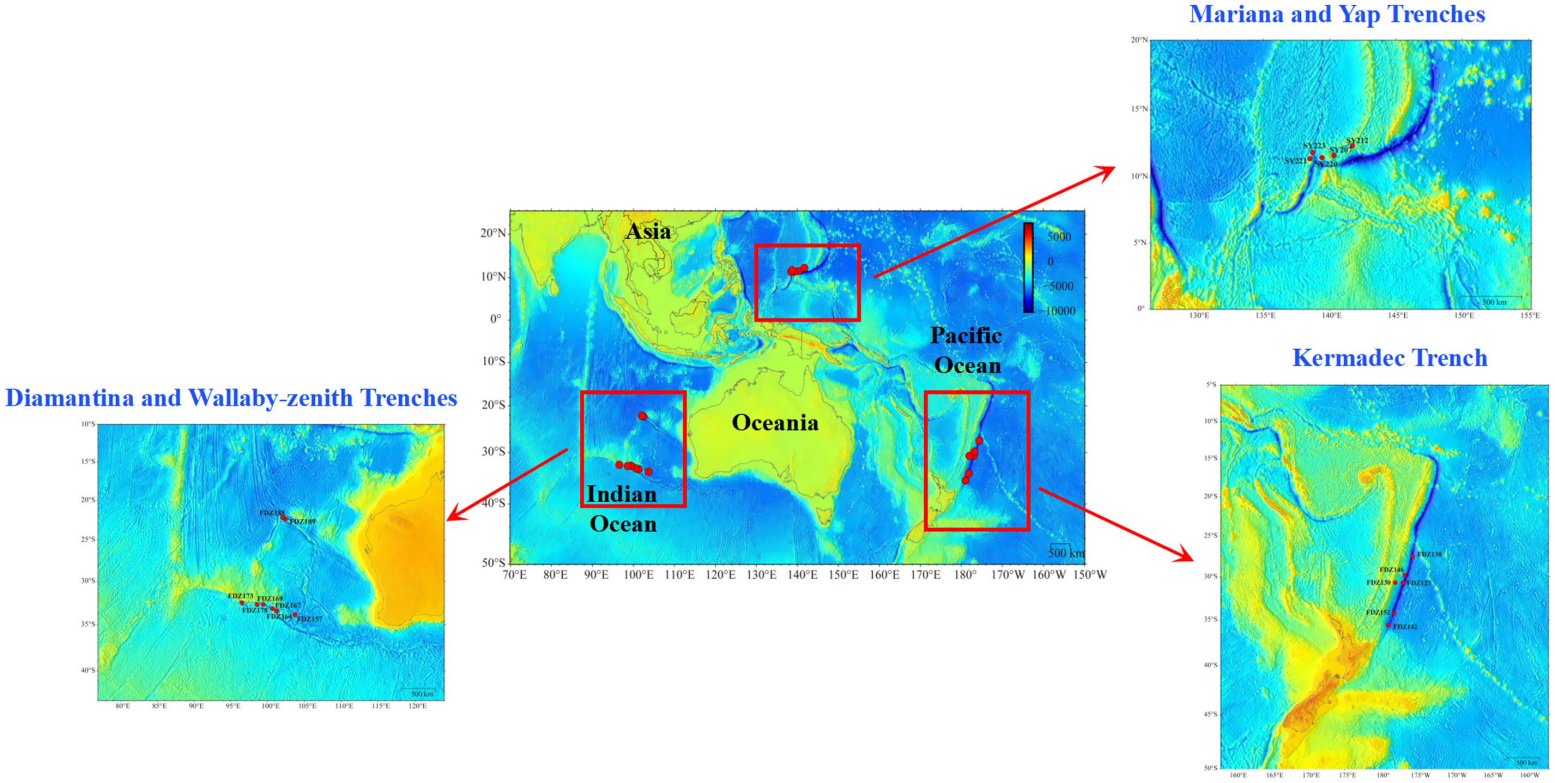
A map showing the sampling locations for the five different trenches.

Pushcore sediment samples were collected from the Yap and Mariana trenches during cruise TS14 on R/V “*Tansuoyihao*” in 2019. Samples from the Kermadec, Diamantina, and Wallaby-Zenith trenches were collected during cruise TS29 on R/V “*Tansuoyihao*” in 2022 (Figure 1). *In situ* hydrographic parameters (location and depth, and so on) were measured with the manned submersibles “*Shenhaiyongshi*” and “*Fendouzhe*,” respectively. The top two layers (i.e., the surface layer of 0–10 cm and the lower layer of 10–20 cm), each 10 cm below the seafloor, were sliced into two fractions. One fraction for subsequent cultivation was stored at 4 °C in the dark, and the other fraction for DNA extraction was stored at −80 °C (Table 1). In total, nine sediment samples were collected from 2,600 meters below sea level (mbsl) to 3,489 mbsl in the MT and YT, and 19 sediment samples were collected from 3,938 to 9,100 mbsl in the KT, DT, and WT.

**Table 1:** Environmental variables of sediment samples collected from different trenches.

Regions	Stns.	TOC (mg/kg)	NO_3_^−^ (mg/kg)	NH_4_^+^ (mg/kg)	TP (mg/kg)
Kermadec Trench (KT)	FDZ127	3,927.95	8.66	1.96	596.88
	FDZ130	3,594.19	1.97	1.42	539.16
	FDZ138	4,038.48	2.17	7.67	953.98
	FDZ142	4,286.11	3.79	2.98	544.33
	FDZ146S	1,507.91^**^	2.04	10.08	934.09
	FDZ146B	1,137.24	1.69	8.48^**^	863.35^*^
	FDZ152S	2,798.291^**^	2.03	6.04	672.71
	FDZ152B	2,206.03	2.48	3.90^**^	849.80^*^
Diamantina Trench (DT)	FDZ157	4,665.22	3.45	4.09	930.00
	FDZ164S	5,741.781^**^	5.15	5.46	889.56
	FDZ164B	3,256.76	3.81	4.69^**^	1176.05^*^
	FDZ167	5,108.78	2.58	6.63	1149.78
	FDZ169	9,998.64	2.29	6.84	837.69
	FDZ173S	8,408.551^*^	3.16	5.86	1029.55
	FDZ173B	8,020.80	1.59	5.41^**^	908.14^*^
	FDZ178	5,176.20	5.12	5.65	1546.62
Wallaby-Zenith Trench (WT)	FDZ188S	17,043.50^#,**^	2.72	6.45	728.86
	FDZ188B	11,218.83^#^	2.10	5.96^**^	902.41^*^
	FDZ189	5,331.73^#^	5.65	5.12	1022.44
Mariana Trench (MT)	SY207	37,760.78^#^	2.35	5.87	870.44
	SY212S	11,604.82^#,**^	3.17	8.21^**^	1009.70
	SY212B	11,076.31^#^	3.47	9.36	1028.12
	SY220S	7,427.14^#,**^	3.06	3.16^**^	376.24
	SY220B	5,824.72^#^	2.42	11.74	453.40
Yap Trench (YT)	SY221S	9,602.35^#,**^	2.62	3.89^**^	209.74
	SY221B	8,352.63^#^	4.07	11.30	267.27
	SY223S	27,440.89^#,**^	3.92	6.94^**^	874.60
	SY223B	7,072.10^#^	2.63	7.05	916.44

S and B represent the 0–10 cm and 11–20 cm sediment layers, respectively. *, **, and # denote *p* < 0.05, *p* < 0.01, and *p* < 0.01, respectively, indicating statistical differences between sediment layers and different trenches.

Approximately 5 g of homogenized sediment was used to measure chemical parameters at the Institute of Mountain Hazards and Environment, Chinese Academy of Sciences (Chengdu, Sichuan, China). The sediment treated with 1 M HCl was analyzed using a colorimetric auto-analyzer (SEAL Analytical AutoAnalyzer 3, Germany) to determine nitrate (NO_3_^−^) and ammonium (NH_4_^+^) concentrations. The content of total organic carbon (TOC) was determined using an elemental analyzer (Elementar Vario Macro Cube, Germany) after drying the sediment at 105 °C (Wang et al., 2016). After treatment with nitric-perchloric acid, the total phosphate (TP) content was measured using the molybdate colorimetric method on a UV-2450 (Shimadzu, Japan; Murphy and Riley, 1962).

### 2.2 Microbial community productivity and respiration

The incubation experiment was conducted onboard in the dark at the *in situ* temperature (4 °C), and the production and respiration samples collected afterward were frozen at −80 °C until laboratory analysis. Total microbial community productivity was quantified using a modified version of the ^3^H-leucine incorporation method described by Knap et al. (1996) under atmospheric pressure (AP). The high hydrostatic pressure (HHP) experimental group was prepared and processed identically to the above, except under *in situ* pressure. The control replicates were prepared and processed in the same way as above, except that a final concentration of 5% trichloroacetic acid (TCA) was added before the addition of the isotope. The ^3^H-leucine activity was measured with a Beckman LS 6500 scintillation counter. Scintillation counts were converted into carbon units according to Simon and Azam (1989).

Total microbial community respiration was measured using the electron transport system (ETS) activity method, a routine technique for estimating respiration rates due to its high sensitivity. Sediment samples were resuspended in seawater and then pre-filtered through a 20 μm nylon mesh before measuring respiration rates. *In vivo* ETS activity was evaluated using 2-(4-iodophenyl)-3-(4-nitrophenyl)-5-(phenyl)tetrazolium chloride (INT) as an electron acceptor to determine the microbial respiration rate (Martínez-García et al., 2009) with AP. The HHP experimental group was prepared and processed identically to the above, except at the *in-situ* pressure. The standard solution (0.02–42 μM) was made by adding the configured INT mother liquor (7.9 mM) to the n-propanol solution. The standard curve was generated from OD values measured at 485 nm using a spectrophotometer. According to the transformation parameters of 12.8 for the INT production rate and O_2_ consumption rate in living cells (Martínez-García et al., 2009), the respiration rate for the total microbial community in each sample was determined using AP and HHP. Oxygen consumption was converted to carbon respiration by assuming that sediment oxygen consumption was 1:1 with organic matter oxidation to CO_2_ (Nakamura, 2003).

Growth efficiency was calculated based on respiration rate and productivity (Warkentin et al., 2011). According to this, we propose the following formula:


PGE=PP/(PP+PR)

In equations, PP and PR stand for prokaryotic productivity and respiration rate, respectively; PGE means prokaryotic growth efficiency.

### 2.3 DNA extraction, PCR amplification, and sequencing

Genomic DNA was extracted from the surface (0–10 cm) and deeper (11–20 cm) sediment layers using the PowerSoil DNA Isolation Kit (QIAGEN Laboratories, Inc., Carlsbad, USA) according to the manufacturer’s protocol. The DNA concentration was determined using a Qubit 2.0 (Life Technologies, USA), and the quality was assessed by gel electrophoresis. The V3-V4 region of the 16S rRNA gene was amplified with universal prokaryotic primers of Uni341F (5′-CCTACGGGNBGCASCAG-3′) and Uni805R (5′-GACTACNVGGGTATCTAATCC-3′; Takahashi et al., 2014). For each sample, a unique barcode pair was used. PCR amplification was carried out in triplicate using the BIO-RAD C1000 Touch Thermal Cycler PCR System in a 20 μl PCR reaction mix, containing 2.0 μl 10 × PCR-MgCl_2_ buffer, 0.5 μl 2.5 mM dNTPs, 0.5 μl MgCl_2_, 0.5 μl forward primer, 0.5 μl reverse primer, 0.2 μl Platinum TaqDNA polymerase, 2.2 μl template DNA, and 13.8 μl ddH_2_O. Thermal cycling was performed at 95 °C for 3 min, followed by 40 cycles at 95 °C for 0.5 min, 53 °C for 45 s, 72 °C for 30 s, and a final extension at 72 °C for 8 min. A negative control using double-distilled water was also performed during amplification to avoid reagent contamination. Paired-end sequencing of the amplicons was performed on an Illumina HiSeq PE250 sequencer (Novogene Co., Ltd., www.novogene.com).

### 2.4 Quantitative PCR

The abundance of 16S rRNA genes in each sample was quantified by real-time quantitative PCR (qPCR) using a StepOnePlus Real-Time PCR system (Applied Biosystems Inc., Carlsbad, CA, USA). Each qPCR reaction comprised 10 μl of 2 × SYBR Premix Ex Taq II (Takara Bio Inc., Shiga, Japan), Uni340F (5′-CCTACGGGRBGCASCAG-3′)/Uni806R (5′-GGACTACNNGGGTATCTAAT-3′) primers (Takai and Horikoshi, 2000), 2 μl of DNA as the template, 0.4 μl of ROX reference dye, and water to make a total volume of 20 μl. The qPCR reactions and calibrations were performed according to a previously described protocol (Takai and Horikoshi, 2000). Briefly, triplicate qPCR reactions were performed for each sample, with efficiencies ranging from 90.20%, and the C_t_ values were calculated as gene copies relative to the standard curve (R^2^ = 0.9958, per gram of wet sediment). As a positive control, a linear plasmid was constructed using the amplified PCR products and a TOPO-TA vector cloning kit (Invitrogen). Gene abundance per sample was determined by multiplying the elution volume by the sediment weight after DNA extraction.

### 2.5 Bioinformatics analysis

After sequencing, barcodes and low-quality sequences were removed using QIIME2 (Caporaso et al., 2010). Chimeras were detected using UCHIME against the SILVA database release 138 (Quast et al., 2013), and singletons were manually removed. The remaining reads were then clustered into Amplicon Sequence Variants (ASV) using DADA2 (Divisive Amplicon Denoising Algorithm; Callahan et al., 2016). Taxonomic assignments of ASVs not affiliated with prokaryotes, as determined from the SILVA database release 138, were further removed (Caporaso et al., 2010). The Shannon index, Simpson index, and richness estimator (Chao1) were calculated based on the Bray–Curtis dissimilarity matrix. Network analysis was conducted to explore co-occurrence patterns within and between taxa in microbial communities. A similarity matrix was first generated using a typical ASV matrix file.

The correlation matrix, along with the *r* and *p* values, was then calculated using corr. test from the “psych” package (Revelle, 2015) in R version 3.5.3. ASVs showing strong and significant correlations (Spearman’s |r| > 0.6 and FDR-adjusted *p* < 0.05) were used to construct networks in Gephi version 0.9.3 (Bastian et al., 2009). Predicted potential carbon metabolic pathways for microbial communities based on the 16S rRNA gene were conducted with FAPROTAX 1.2.12 (Louca et al., 2016). The 16S rRNA gene abundance, production rate, respiration rate, and growth efficiency of the prokaryotic community were plotted using SigmaPlot 12.0 (Systat Software, Inc., SigmaPlot for Windows). Values of *p* < 0.05 and *p* < 0.01 were considered indicators of different levels of statistical significance.

### 2.6 Statistical analysis

Non-metric multidimensional scaling (NMDS), based on the Bray–Curtis similarity index, was calculated using PRIMER 5 (Plymouth Marine Laboratory, West Hoe, Plymouth, UK; Clarke and Warwick, 2001) to visualize the distribution patterns of microbial communities. An analysis of similarities (ANOSIM), based on ASV relative abundance, was conducted in Paleontological Statistics (PAST) version 3 (Hammer et al., 2001) to test for significant differences in microbial community composition among sampling sites. The Pearson correlation coefficients between environmental variables and the metabolic features of the microbial communities were calculated separately using GraphPad Prism version 8.0 (GraphPad Software, Inc., San Diego, CA, USA), after square-root transformation of the data. Values of *p* < 0.05 and *p* < 0.01 were considered to indicate different levels of statistical significance.

Since the length of axis 1 of detrended correspondence analysis (DCA) > 3.0, canonical correspondence analysis (CCA) was performed to analyze the associations between prokaryotic communities and environmental factors using CANOCO v5.0 software (Šmilauer and Lepš, 2014). In addition. Welch’s *t*-test was used to identify differences in the relative abundance of microbial communities, employing the confidence interval method in the Statistical Analysis of Metagenomic Profiles (STAMP, Parks et al., 2014) software package (version 2.1.3). Moreover, a *t*-test was conducted to compare nutrient concentrations after pooling sediment samples from each group.

## 3 Results

### 3.1 Geochemical characterization of the samples

Generally, the TOC content of sediments was significantly higher in the MT-YT than in the KT-DT-WT (*t*-test, *p* < 0.01), which might be due to the much lower sampling depth in the former. Furthermore, the TOC content of surface sediments was significantly higher than that of deeper sediments (*t*-test, *p* < 0.01). In the KT, DT, and WT, the TOC and NO_3_^−^ levels of sediments in DT and MT were significantly higher than those of sediments in KT (*t*-test, *p* < 0.01). A lower concentration of NH_4_^+^ was found in the surface layer of the MT and YT, whereas in the deeper layers of the KT, DT, and WT. TP content was significantly higher in the KT, DT, and WT than in their counterparts (*t*-test, *p* < 0.05; Table 1).

### 3.2 Community composition, diversity, and gene abundances

A total of 1,551,838 sequences and 1,541 ASVs were generated by the microbial communities, with the highest and lowest numbers of ASVs found in the deeper sediment layers at stations (Stns.) FDZ188 and FDZ173 in the KT, DT, and WT (Table S1). The highest and lowest numbers of ASVs were found in the deeper sediment layer at station (Stn.) SY220 and the surface sediment layer at Stn. SY223 in the MT and YT, respectively. The number of ASVs in sediments was also higher in the deeper layers than in the surface layers, except at Stns. FDZ152 and SY212.

**Figure 2:**
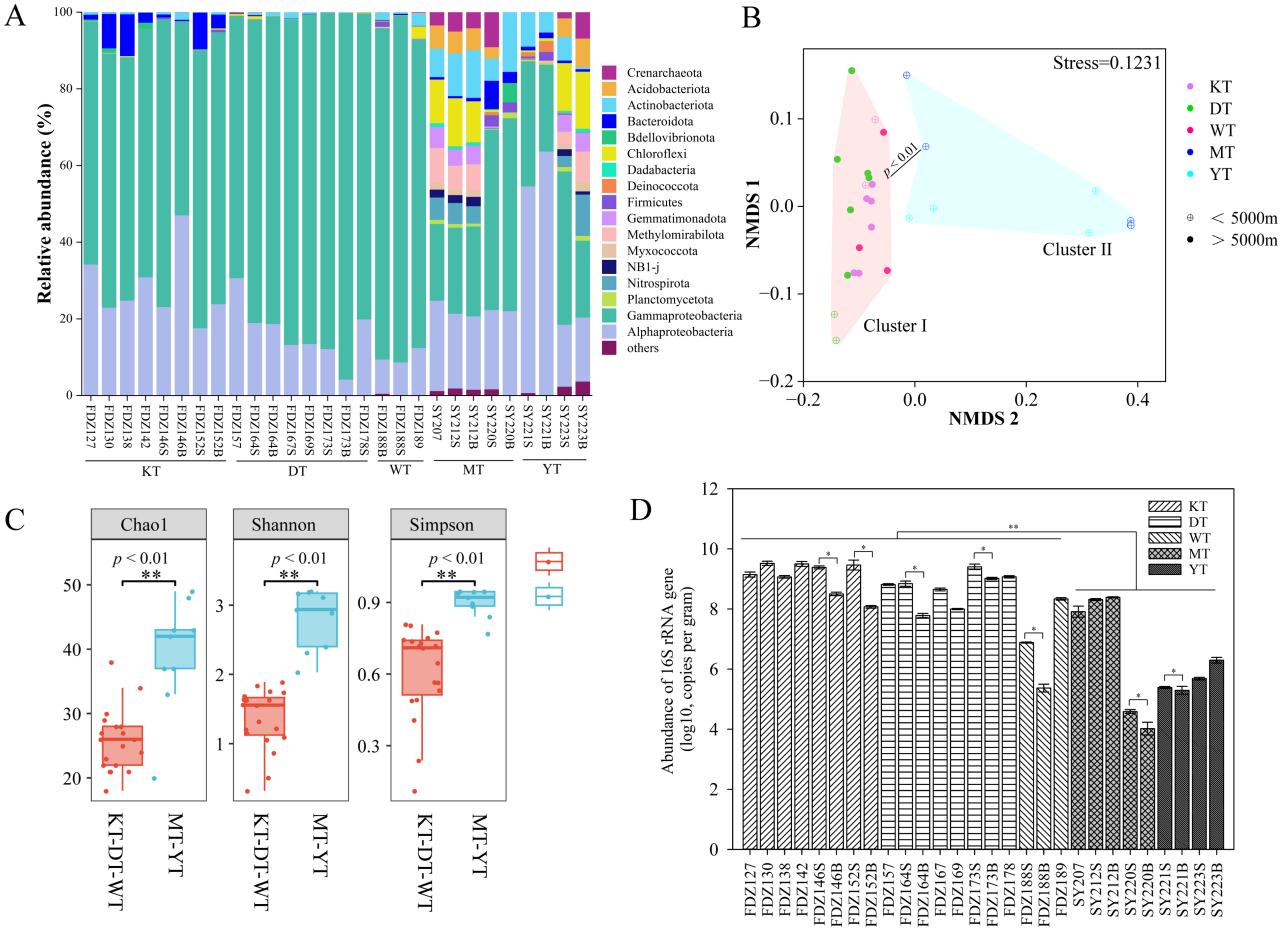
Microbes across the five different trenches. Microbial community structure of various sediment samples at the order level **(A)**. Non-metric multidimensional scaling (NMDS) plot of microbial communities based on all the ASVs **(B)**. The Chao1, Shannon, and Simpson diversity indices in sediment samples; values of *p* < 0.01 were considered to indicate a different level of statistical significance **(C)**. Abundance of microbial 16S rRNA gene with error bars representing standard deviation **(D)**. S and B stand for 0–10 cm and 11–20 cm depths of sediment, respectively. * *p* < 0.05, ** *p* < 0.01.

In terms of community composition, Nitrososphaeria in the Crenarchaeota was the dominant archaeal class across all samples (Figure 2A). Nitrososphaeria showed significantly higher proportions in the MT and YT than in its counterpart (*t*-test, *p* < 0.05). As for bacteria, Alphaproteobacteria and Gammaproteobacteria were the dominant classes in the KT, DT, and WT, accounting for more than 90% of the total retrieved sequences, while Alphaproteobacteria, Gammaproteobacteria, Actinobacteria, and Bacteroidia were dominant in the MT and YT (Figure 2A). NMDS analysis demonstrated that the microbial communities in cluster I (KT-DT-WT) and cluster II (MT-YT) were significantly distinct from each other (ANOSIM, *p* < 0.01, Figure 2B).

Moreover, within each individual trench (KT or DT), microbial communities from sediments below 5,000 m were significantly different from those above 5,000 m (Figure S1). Significantly higher Chao1, Shannon, and Simpson diversity indices were found in the group MT-YT (Kruskal–Wallis test, *p* < 0.01, Figure 2C). According to the STAMP analysis, the indicative groups were primarily affiliated with *Oceanospirillales*, *Alteromonadales,* and *Pseudomonadales* in the KT, DT, and WT trenches, while they were mainly associated with *Rhizobiales* and *Actinomarinales* in the MT and YT trenches (Welch’s *t*-test, *p* < 0.05, Figure S2). Comparatively, the 16S rRNA gene abundance was significantly higher in the KT, DT, and WT (*t*-test, *p* < 0.05, Figure 2D) and in the surface layer, except for Stns. SY212 and SY223 (Figure 2D).

### 3.3 Potential functions and co-occurrence network of microbial communities

**Figure 3:**
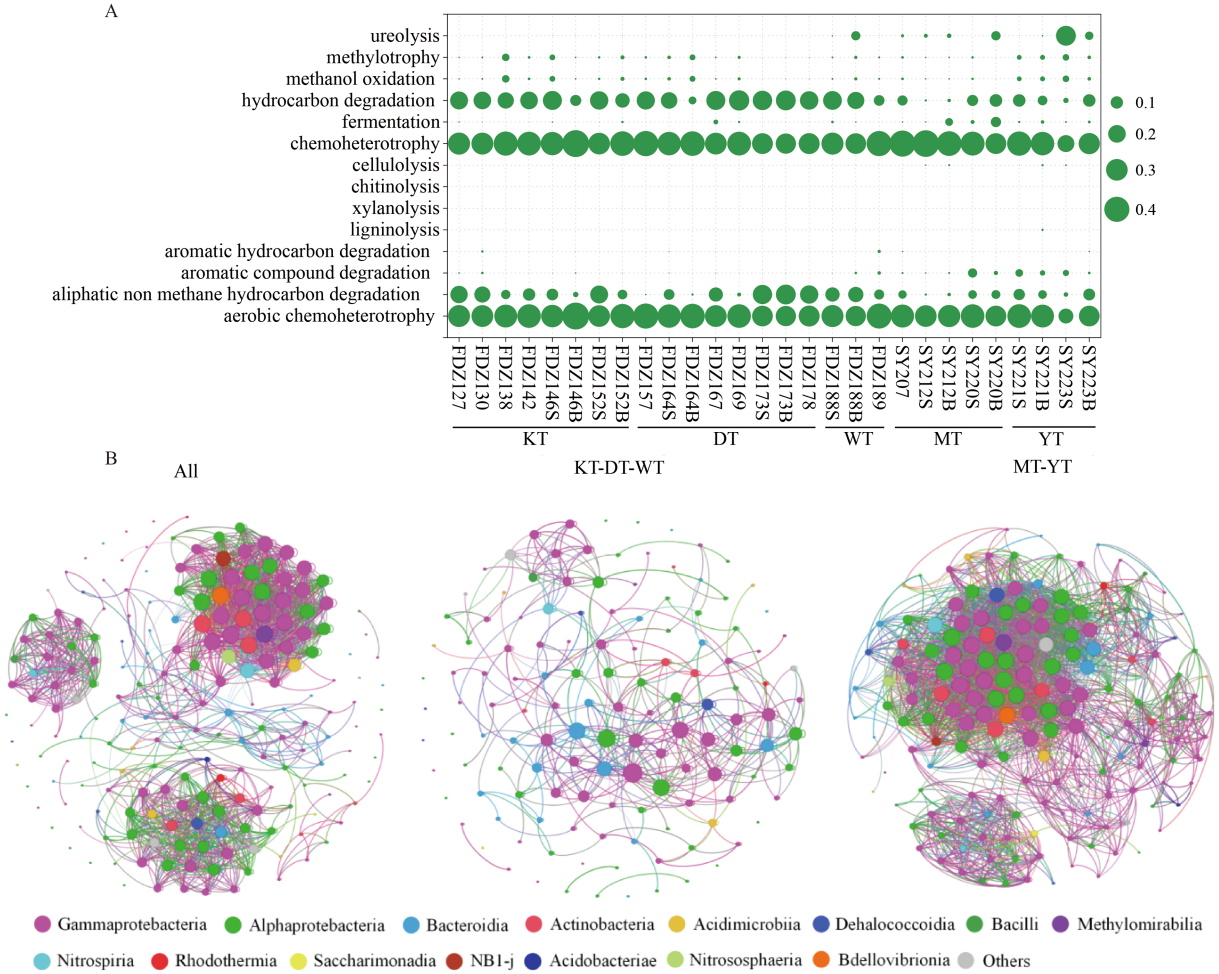
Microbial carbon potential functions and co-occurrence networks. A summary of the mean abundance of functional predictions related to carbon metabolism for microbial communities from the sediment sample **(A)**. S and B represent the 0–10 cm and 11–20 cm depths of sediment, respectively. Network analysis of the top 200 most abundant ASVs across all sediment samples **(B)**. The network shows relationships between co-occurring ecosystems; edges represent co-occurrence relationships with a correlation threshold of 0.6; and nodes represent microbial taxa.

Potential functions related to carbon metabolism were predicted from the 16S rRNA genes using FAPROTAX (Figure 3A). The processes of hydrocarbon degradation, chemoheterotrophy, aliphatic non-methane hydrocarbon degradation, and aerobic chemoheterotrophy were significantly enriched (*t*-test, *p* < 0.01). The abundance of these potential functions in the KT, DT, and WT was significantly higher than in their counterparts (*t*-test, *p* < 0.05).

To elucidate the interactions between different microbial groups in the sediments, network analyses were conducted based on the top 200 ASVs (Figure 3B). The resulting network consisted of 200 nodes and 1,756 links for all samples, with 98.5% of the connections positively correlated and 1.5% negatively correlated among these links. In the KT, DT, and WT, the network consisted of 133 nodes and 458 links, with 96.3% of the connections positively correlated. In the MT and YT, the network consisted of 171 nodes and 3,002 links, and 67.3% of the connections were positively correlated (Figure 3B). Overall, positive connections dominated interactions among microbial taxa in the sediments. However, more negative connections were observed in the MT and YT. Negative correlations were found mainly between *Rhodobacterales* and *Alteromonadales*, *Pseudomonadales* and *Bacteriovoracales*, as well as *Sphingomonadales* and *Alteromonadales* in the KT, DT, and WT, while mainly between *Rhizobiales* and *Burkholderiales*, and *Pseudomonadales* and *Alteromonadales* in the MT and YT (Figure 3B). As for the network of the rare microbial taxa, all the connections were positively correlated (Figure S3).

### 3.4 Metabolic characteristics of microbial communities

**Figure 4:**
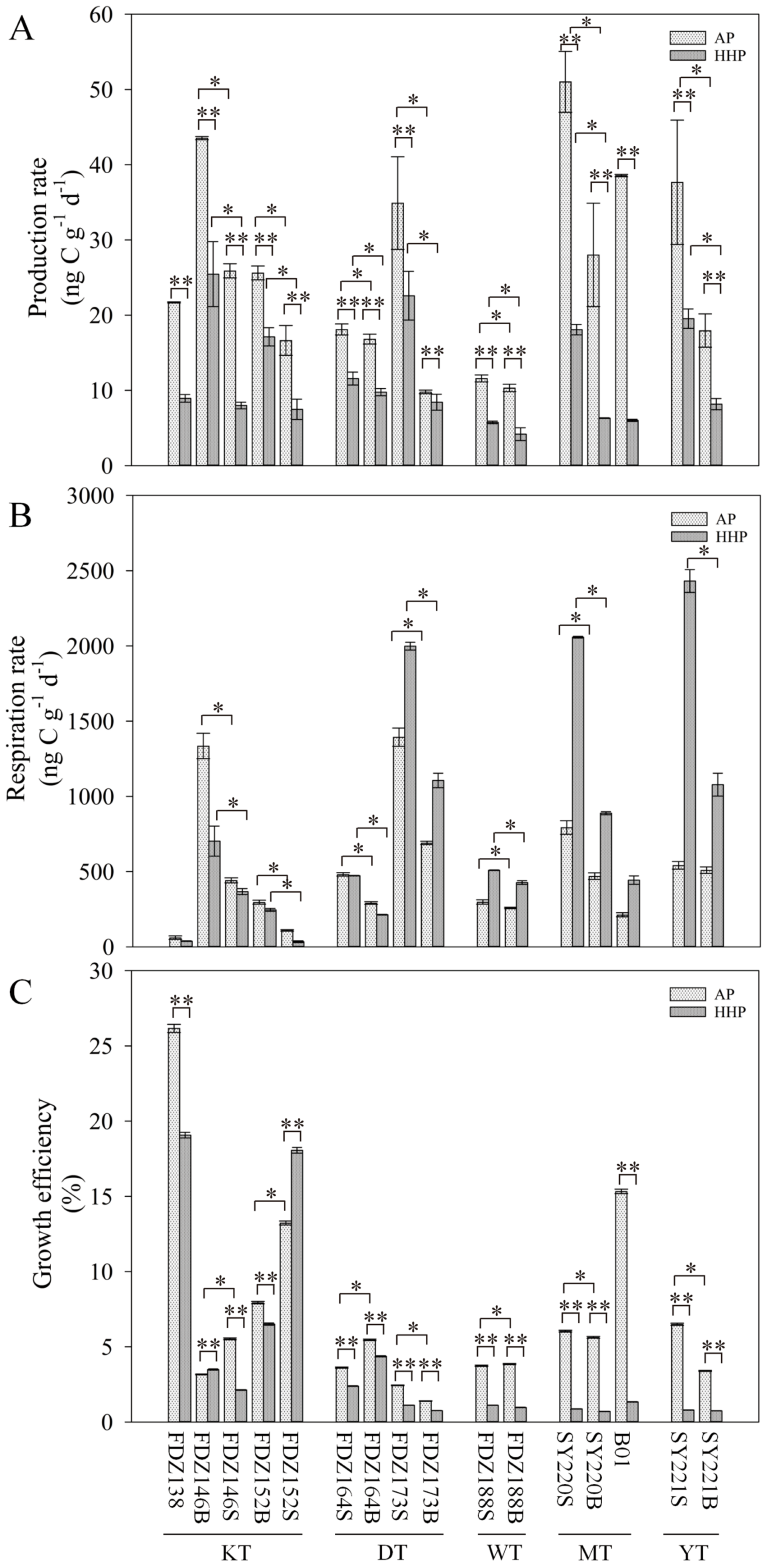
Effects of pressure on microbial metabolic rates. The production rate **(A)**, respiration rates **(B)**, and growth efficiency **(C)** of microbial communities under atmospheric pressure and high hydrostatic pressure with wet sediments. S and B represent 0–10 cm and 11–20 cm depths of sediment, respectively. *t*-test: *p < 0.05, ** *p* < 0.01.

The production rates did not differ significantly between the two clusters at the same pressure. However, production rates were significantly higher under AP than those under HHP (*t*-test, *p* < 0.01, Figure 4A). On a vertical scale, the production and respiration rates were significantly higher in surface layers than those in deeper layers under the same pressure (*t*-test, *p* < 0.05, Figures 4A, B). The respiration rates were significantly higher in the MT and YT than in the KT, DT, and WT under AP (*t*-test, *p* < 0.05, Figure 4B), while there was no significant difference between the two clusters under HHP (Figure 4B). However, significantly higher respiration rates under HHP were found in the MT and YT (*t*-test, *p* < 0.01, Figure 4B), but significantly lower respiration rates under HHP were found in the KT, DT, and WT (*t*-test, *p* < 0.01, Figure 4B), except for Stns FDZ173 and FDZ188. The PGEs were significantly higher under AP than under HHP (*t*-test, *p* < 0.01, Figure 4C). On a vertical scale, the PGEs were higher in surface layers than in deeper layers under AP, while no significant difference was observed under HHP (Figure 4C).

### 3.5 Environmental effects and correlation analysis

**Figure 5:**
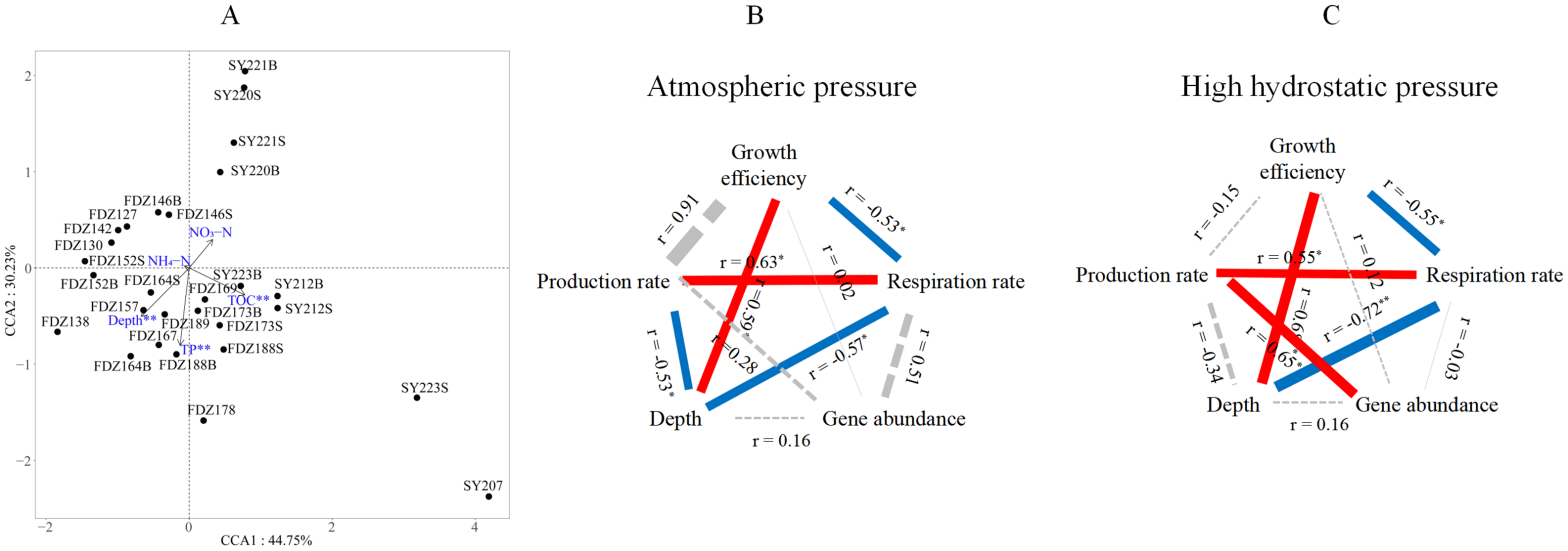
Microbial community structure and its environmental drivers. Redundancy analysis of community structure and environmental factors of microbial communities based on all the ASVs **(A)**. S and B stand for 0–10 cm and 11–20 cm depths of sediment, respectively. Pearson correlations among gene abundance, production rate, respiration rate, and growth efficiency with associated key environmental factors under atmospheric pressure **(B)** and high hydrostatic pressure **(C)**. The solid red and blue lines represent significantly positive and negative correlations, respectively, and the dashed black line represents a statistically non-significant correlation. The line thickness reflects the absolute range of the correlation coefficient. * *p* < 0.05, ** *p* < 0.01.

Since microbial communities were retrieved from the two clusters, as demonstrated by the NMDS analysis, environmental effects on these communities were further analyzed using CCA. After removing factors with VIF > 10, five environmental parameters (i.e., depth, TOC, TP, NH_4_-N, and NH_3_-N) were used for CCA analysis. Among them, depth, TOC, and TP had significant effects on the microbial community (999 permutation test, *p* < 0.01; Figure 5A).

Pearson correlations among gene abundance, metabolic rates, and key environmental factors in sediments were also conducted (Figures 5B,C). Only depth had significant correlations with microbial metabolic features in the sediments. Among metabolic features, the respiration rate showed a consistently negative correlation with depth under AP (R = −0.57, *p* < 0.05) and HHP (R = −0.72, *p* < 0.01). Growth efficiency was always significantly correlated with depth under AP (R = 0.59, *p* < 0.05) and HHP (R = 0.68, *p* < 0.01). The production rate showed a negative correlation with depth only under AP (R = −0.53, *p* < 0.01, Figure 5B).

## 4 Discussions

### 4.1 Heterogeneity of microbial communities and microbial metabolic characteristics among trenches

Distinct community structures among the five trenches suggest that the geographical scale is an essential driver of the biogeographic pattern of microbes (Martiny et al., 2006; Hanson et al., 2012). The lack of a significant difference between the MT and YT is very likely due to ocean circulation, as a westward-flowing lower circumpolar deep water penetrates the Philippine Sea through the Yap-Mariana Junction at depths below 4,000 m (Zhou et al., 2022), which may have facilitated connectivity between microbial communities in the trenches. A higher diversity of the bacterial communities than that of the archaeal communities has been reported previously in each trench (Liu et al., 2023; Zhang and Jing, 2024).

The major microbial groups revealed in the present study were consistent with the large-scale metagenomic survey of hadal sediments by Xiao et al. (2025). Alteromonadaceae were key degraders of complex organic matter via extracellular enzymes (Hoffmann et al., 2017), while Oceanospirillaceae were crucial for degrading recalcitrant hydrocarbons in trench sediments (Liu et al., 2019). Rhodobacteraceae played an important role in the carbon cycle by degrading polysaccharides and transparent exopolymer particles (Taylor and Cunliffe, 2017). These microbial groups were capable of utilizing different carbon sources, possibly occupying different niches (Teske et al., 2011), and cooperatively drove the carbon cycles in different trenches.

Significantly higher production rates, respiration rates, and growth efficiencies of microbial communities occurred in the surface layers compared to the deeper layers; this disparity could be partially attributed to variations in dissolved oxygen and the accessibility of organic carbon (Paerl et al., 2002; Li et al., 2023), because higher fluxes of “sea snow” from the upper water column and more organic carbon accumulated in the surface sediments.

There is a negative correlation between sampling depth and microbial production and growth efficiency under ambient pressure, highlighting increased selective pressure and reduced resource availability at greater depths (Peoples et al., 2018, 2019; Wang et al., 2022). Similarly, the production rates in trenches were considerably lower than those observed in the Mediterranean (Amalfitano et al., 2008) and the Northeast Atlantic (Dixon and Turley, 2001), and they were also lower than the average benthic microbial production (Cole et al., 1988). The respiration rates were lower than those observed in Omura Bay (Wada et al., 2012), the East China Sea (Song et al., 2016), the northern Gulf of Mexico (Rowe et al., 2002), and the Seto Inland Sea (Nakamura, 2003).

It is notable that the lower growth efficiency was observed in the MT and YT sediments, where microbial communities exhibited higher respiration and production rates. Carbon oxidation required less energy (Røy et al., 2012). Therefore, microbes may be more inclined toward respiration than production. While the TOC content of sediments was significantly higher in the MT and YT than in the KT, DT, and WT, this might contribute to higher respiration and production rates in the former. Furthermore, in the MT and YT, a greater proportion of nitrite-oxidizing Nitrospira is present—possibly driven by nitrification—which might explain the higher respiration rates observed (Leung et al., 2022).

Concurrently, organic carbon availability was a primary driver of microbial metabolism, with higher TOC in MT and YT supporting greater respiratory activity and carbon turnover than in KT and DT, as indicated by a positive correlation between TOC and respiration rate (Glud et al., 2013). The elevated particulate organic matter flux in the Mariana and Yap Trenches stimulated heterotrophic degradation and nitrification, consuming NH_4_^+^ in surface sediments (Glud et al., 2013; Liu et al., 2023). Conversely, in KT, DT, and WT, extremely slow microbial activity and the cessation of nitrification below the redox interface led to the accumulation of NH_4_^+^ in deeper layers (Jørgensen and Boetius, 2007).

### 4.2 Pressure impact and ecology implications

Deep-sea microbial communities are exposed to high pressure, which has variable effects on prokaryotes depending on whether they are piezophilic, piezotolerant, or piezosensitive. By far, the impacts of hydrostatic pressure on microbial metabolic processes remain poorly understood. This study revealed that the production rates were significantly higher under AP than under HHP， indicating the passive effect of pressure. Deep-sea microbial heterotrophic activity under atmospheric pressure was suppressed to about one-third of that under *in situ* hydrostatic pressure (Amano et al., 2022). The MT and YT exhibited significantly higher respiration rates under AP than the KT, DT, and WT, which might be attributed to their higher TOC content and distinct microbial community structures, dominated by heterotrophic taxa such as Gammaproteobacteria and Bacteroidia.

In contrast, the KT and DT showed lower respiration but comparable production rates under HHP, suggesting trench-specific adaptations to pressure and organic matter availability. These variations in respiration and production directly influenced prokaryotic growth efficiency, which was consistently lower under HHP across all trenches, indicating a general shift in carbon allocation from biomass production to maintenance respiration under *in situ* conditions. This aligned with previous findings that HHP could reduce the rate of aerobic oxidation of fatty acids in the TCA cycle (Scoma et al., 2019). Consequently, the high degradation rates exhibited by obligate hydrocarbon degraders might become disadvantageous at elevated HHP (Schedler et al., 2014; Amano et al., 2022).

Similarly, the PGEs were significantly higher under AP than under HHP, and the PGE values ranged from 1 to 19% under HHP in this study. Because most previous studies neglected the pressure effect and were conducted solely at atmospheric pressure (Herndl and Reinthaler, 2013; Amano et al., 2022), heterotrophic biomass production by deep-sea prokaryotes has been overestimated. According to the method proposed by Amano et al. (2022), the estimated carbon demand derived from *in situ* pressure-activity measurements and the particulate organic carbon supply were largely balanced. It was very likely that biomass production and respiration of the bulk prokaryotic community were reduced in proportion to the *in situ* pressure.

We further showed that PGEs and hydrostatic pressure in prokaryotes were significantly negatively correlated (Amano et al., 2022), as hydrostatic pressure can significantly inhibit the heterotrophic activity of deep-sea prokaryotic communities. On the other hand, the effects of pressure on respiration rates were inconsistent; this might reflect the influence of hydrostatic pressure on the physiological, metabolic, and enzymatic activities of microorganisms in hadal trenches (Xiao et al., 2021). In the presence of HHP, microorganisms prefer anaerobic metabolisms over aerobic respiration as a “common adaptation strategy” since the former could cause less intracellular oxidative stress (Xiao and Zhang, 2014), resulting in a shift of the redox gradient, leading to a greater reaction of Gibbs free energy being generated from denitrification compared to that under AP (Zhang et al., 2015).

Under reduced pressure, the metabolic activity of deep-sea piezosensitive microbes can increase by more than 100-fold, potentially leading to a significant overestimation of *in situ* metabolic rates (Tamburini et al., 2013; Amano et al., 2022). Consequently, high pressure generally suppresses microbial activity, suggesting that the deep-sea carbon cycle might be slower than previously thought, with implications for global carbon budgets (Burd et al., 2010; Herndl and Reinthaler, 2013; Amano et al., 2022). Our functional predictions indicated enrichment for hydrocarbon degradation and chemoheterotrophy in the KT-DT-WT cluster, consistent with the genetic potential reported in trench metagenomes (Xiao et al., 2025). Crucially, our physiological measurements demonstrated that this high genetic potential did not translate into efficient biomass production *in situ*. We provided direct evidence that HHP severely suppresses PGE, shunting carbon flow from growth to maintenance respiration—a key constraint on carbon cycling that genomic data alone could not reveal—underscoring the necessity of coupling genomic and physiological approaches.

## 5 Conclusion

This study conducted a comprehensive investigation of the microbial community structure and metabolic characteristics of sediments from five global trenches. Distinct microbial community structures and diversities existed among the trenches due to geographic distance and trench-specific geochemical characteristics. The activities of radioactive isotopes and the electron transport system were applied to understand the processes and efficiency of microbial-driven carbon cycles. By integrating microbial community analysis with direct measurements of metabolic rates under simulated *in situ* pressure, this study revealed that the growth efficiency of microbial communities across the five trenches was significantly constrained by HHP. This physiological constraint, which led to a preferential use of carbon for respiration over biosynthesis, was a fundamental factor regulating the efficiency of the microbial carbon pump in the deep-sea biosphere, providing a crucial physiological foundation for the functional genomic study (Xiao et al., 2025). Future investigations into bacteria and archaea separately would help us gain a more comprehensive understanding of carbon utilization and assimilation by microbes in the deep-sea biosphere.

## Data Availability

All of the 16S rRNA gene sequences obtained from this study have been deposited in the National Center for Biotechnology Information (NCBI) Sequence Read Archive (SRA) under the accession number PRJNA1124523.
