# Supplementary material for: Microbial community structure and metabolic characteristics in sediments from five different deep-sea trenches
Source: Front Microbiol. 2025 Dec 3;16:1676738. doi: 10.3389/fmicb.2025.1676738 (PMC12709017; doi:10.3389/fmicb.2025.1676738)
Supplement: Supplementary file 1 [file Data_Sheet_1.doc]

**Table S1** The sequence information of sediment samples collected from five different trenches. S and B represent the 0-10cm and 11-20cm sediment layers, respectively. mbsl stands for meters below sea level.

| **Regions** | **Stns.** | **Longitude** | **Latitude** | **mbsl**  **(m)** | **Original**  **Reads** | **Quality**  **Reads** | **ASVs** |
| --- | --- | --- | --- | --- | --- | --- | --- |
| Kermadec Trench  (KT) | FDZ127 | 176.91°W | 30.69°S | 7,600 | 114,341 | 60,842 | 93 |
|  | FDZ130 | 178.04°W | 30.69°S | 5,861 | 102,078 | 42,973 | 143 |
|  | FDZ138 | 175.64°W | 27.54°S | 9,100 | 101,956 | 40,236 | 78 |
|  | FDZ142 | 179.00°W | 35.57°S | 6,471 | 103,817 | 41,234 | 99 |
|  | FDZ146S | 176.76°W | 29.79°S | 3,983 | 103,335 | 41,325 | 110 |
|  | FDZ146B | 176.76°W | 29.79°S | 3,983 | 101,300 | 50,090 | 87 |
|  | FDZ152S | 178.15°W | 34.23°S | 6,969 | 101,208 | 49,870 | 105 |
|  | FDZ152B | 178.15°W | 34.23°S | 6,969 | 101,534 | 44,818 | 117 |
| Diamantina Trench  (DT) | FDZ157 | 103.74°E | 33.87°S | 6,205 | 115,462 | 55,593 | 93 |
|  | FDZ164S | 101.26°E | 33.44°S | 6,792 | 111,920 | 47,188 | 85 |
|  | FDZ164B | 101.26°E | 33.44°S | 6,792 | 105,961 | 49,990 | 66 |
|  | FDZ167 | 100.65°E | 33.17°S | 5,953 | 105,961 | 49,990 | 96 |
|  | FDZ169 | 99.42°E | 32.69°S | 5,154 | 113,418 | 63,719 | 87 |
|  | FDZ173S | 96.53°E | 32.50°S | 4,625 | 101,986 | 72,593 | 95 |
|  | FDZ173B | 96.53°E | 32.50°S | 4,625 | 99,056 | 80,526 | 54 |
|  | FDZ178 | 98.58°E | 32.72°S | 5,677 | 100,900 | 61,683 | 84 |
| Wallaby-Zenith Trench  (WT) | FDZ188S | 102.45°E | 22.37°S | 6,671 | 102,134 | 56,768 | 114 |
|  | FDZ188B | 102.45°E | 22.37°S | 6,671 | 100,718 | 62,567 | 148 |
|  | FDZ189 | 102.08°E | 22.17°S | 6,491 | 99,012 | 43,541 | 131 |
| Mariana Trench  (MT) | SY207 | 140.32°E | 11.61°N | 3,279 | 103,230 | 41,233 | 549 |
|  | SY212S | 141.72°E | 12.32°N | 3,489 | 99,304 | 41,895 | 561 |
|  | SY212B | 141.72°E | 12.32°N | 3,489 | 99,056 | 38,926 | 558 |
|  | SY220S | 139.41°E | 11.44°N | 2,600 | 101,645 | 65,648 | 113 |
|  | SY220B | 139.41°E | 11.44°N | 2,600 | 114,547 | 98,636 | 43 |
| Yap Trench  (YT) | SY221S | 138.52°E | 11.37°N | 2,844 | 102,496 | 55,863 | 137 |
|  | SY221B | 138.52°E | 11.37°N | 2,844 | 113,679 | 89,212 | 176 |
|  | SY223S | 138.72°E | 11.82°N | 3,231 | 99,050 | 49,213 | 600 |
|  | SY223B | 138.72°E | 11.82°N | 3,231 | 99,983 | 55,666 | 572 |

**

**

**Fig. S1** Non-metric multidimensional scaling (NMDS) plot of microbial communities based on all the ASVs of Kermadec Trench (KT) and Diamantina Trench (DT).

**
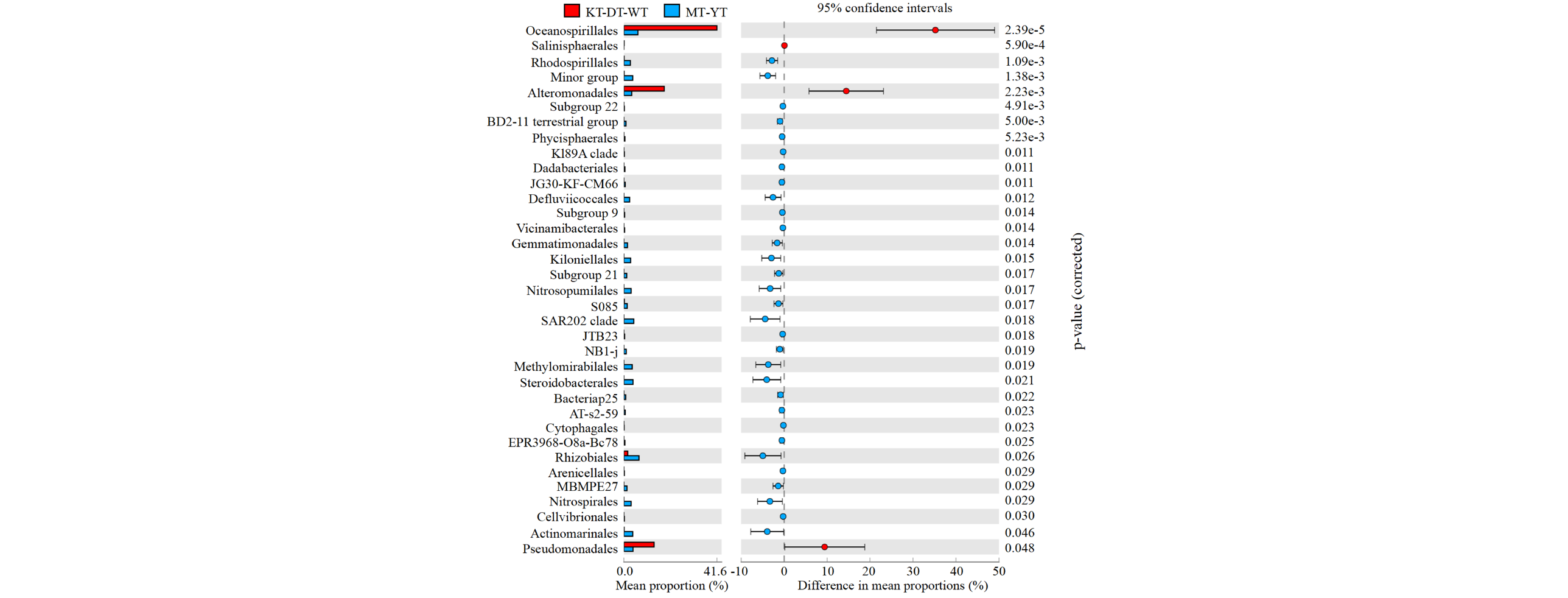
**

**Fig. S2** Extended error plot for microbial communities of the sediments visualized through STAMP software. Mean proportions in different categories were displayed in the left bar graph. The colored circles (brown and blue) showed the 95% confidence intervals calculated using the Welch’s t-test.

**
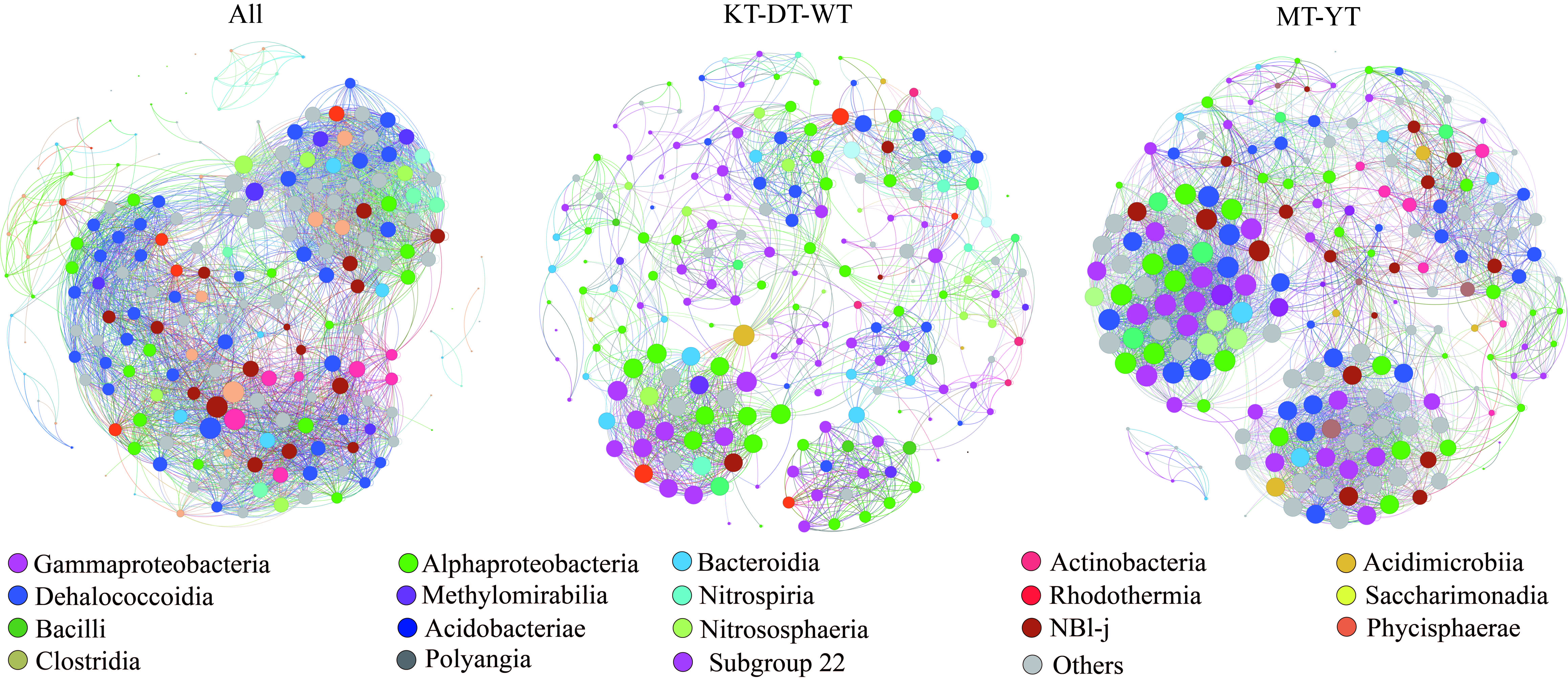
**

**Fig. S3** The networks analysis based on the rare 200 ASVs in all sediment samples. The network represents relationships between co-occurring ecosystems, the edges represent co-occurrence relationships consistent at the 0.6 correlation level, and the nodes represent microbial taxa.
